# HBV Infection and Host Interactions: The Role in Viral Persistence and Oncogenesis

**DOI:** 10.3390/ijms24087651

**Published:** 2023-04-21

**Authors:** Riccardo Nevola, Domenico Beccia, Valerio Rosato, Rachele Ruocco, Davide Mastrocinque, Angela Villani, Pasquale Perillo, Simona Imbriani, Augusto Delle Femine, Livio Criscuolo, Maria Alfano, Marco La Montagna, Antonio Russo, Raffaele Marfella, Domenico Cozzolino, Ferdinando Carlo Sasso, Luca Rinaldi, Aldo Marrone, Luigi Elio Adinolfi, Ernesto Claar

**Affiliations:** 1 Liver Unit, Ospedale Evangelico Betania, 80147 Naples, Italy; valeriorosato@gmail.com (V.R.); davidemastrocinque4@gmail.com (D.M.); pasqualeperillo@hotmail.it (P.P.); ernestoclaar@gmail.com (E.C.); 2 Department of Advanced Medical and Surgical Sciences, University of Campania “Luigi Vanvitelli”, 80138 Naples, Italy; domenico_beccia@yahoo.it (D.B.); rachele.ruocco@yahoo.com (R.R.); angelavill92@gmail.com (A.V.); simo.imbriani@gmail.com (S.I.); gudf96@gmail.com (A.D.F.); livcriscuolo@gmail.com (L.C.); maria.alfano2@libero.it (M.A.); m.lamontagna92@libero.it (M.L.M.); raffaele.marfella@unicampania.it (R.M.); domenico.cozzolino@unicampania.it (D.C.); ferdinandocarlo.sasso@unicampania.it (F.C.S.); luca.rinaldi@unicampania.it (L.R.); aldo.marrone@unicampania.it (A.M.); luigielio.adinolfi@unicampania.it (L.E.A.); 3 Department of Mental Health and Public Medicine, University of Campania “Luigi Vanvitelli”, 80138 Naples, Italy; antonio.russo2@unicampania.it

**Keywords:** HBV, host interaction, liver, cirrhosis, oncogenesis, hepatocellular carcinoma

## Abstract

Hepatitis B virus (HBV) is a major cause of chronic hepatitis, liver cirrhosis, and hepatocellular carcinoma. Despite the advent of vaccines and potent antiviral agents able to suppress viral replication, recovery from chronic HBV infection is still an extremely difficult goal to achieve. Complex interactions between virus and host are responsible for HBV persistence and the risk of oncogenesis. Through multiple pathways, HBV is able to silence both innate and adaptive immunological responses and become out of control. Furthermore, the integration of the viral genome into that of the host and the production of covalently closed circular DNA (cccDNA) represent reservoirs of viral persistence and account for the difficult eradication of the infection. An adequate knowledge of the virus–host interaction mechanisms responsible for viral persistence and the risk of hepatocarcinogenesis is necessary for the development of functional cures for chronic HBV infection. The purpose of this review is, therefore, to analyze how interactions between HBV and host concur in the mechanisms of infection, persistence, and oncogenesis and what are the implications and the therapeutic perspectives that follow.

## 1. Introduction

Despite the availability of vaccines and effective therapies in suppressing the viral load and preventing its transmission, chronic hepatitis B virus (HBV) infection remains a serious world healthcare issue, affecting more than 292 million people worldwide, with an estimated global prevalence of about 4% [1], with different distribution and prevalence in relation to the geographical area, the diffusion of vaccination, and the risk factors [1,2,3]. Chronic HBV infection is one of the major causes of chronic hepatitis, which may lead to cirrhosis and decompensated liver disease [4]. Beyond the capacity to induce liver and systemic damage [5], HBV has a significant oncogenic power, which is linked both to the ability to cause cirrhosis and to viral-induced genetic changes [6]. The course and outcomes of the infection depend on several virus–host interactions, responsible for the tendency to become chronic and to potentially generate a tumor, in particular hepatocellular carcinoma (HCC) [7]. Several mechanisms have been hypothesized through which HBV would be able to escape its host’s defense, integrate its DNA, and induce changes in the host. HBV–host interactions are responsible for the inability of current therapies to eradicate the infection. An adequate knowledge of these mechanisms appears crucial in order to identify new therapeutic targets and to overcome the limits of existing treatments.

The purpose of this review is to examine the role of interactions between virus and host in the mechanisms of infection, viral persistence, and oncogenesis, as well as to analyze the therapeutic implications deriving from them.

## 2. From Infection to Viral Persistence

HBV is a virus belonging to the *Hepadnaviridae* family, characterized by an incomplete double-stranded circular DNA included in an enveloped virion [8]. It can be repaired by an endogenous DNA-polymerase, which can incorporate nucleotides into the genome. Transmission occurs parenterally, and we can find the virus in potentially every body fluid, with a higher concentration in the blood and exudates and a lower concentration in saliva, semen, and vaginal secretions [9].

### 2.1. HBV Replication in the Host Cells

**Figure 1:**
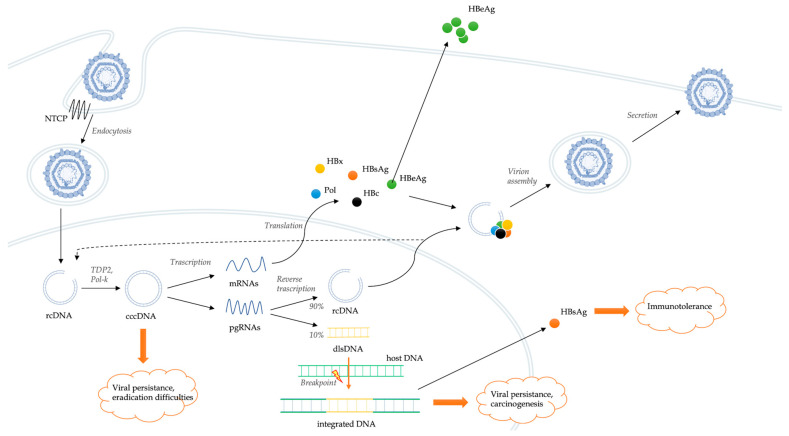
Schematic representation of the HBV life cycle and mechanisms of genomic integration.

HBV entry into the cell is mediated by a low-specificity binding between hepatitis B surface antigen (HBsAg) and heparan sulfate proteoglycans (HSPGs) present on the surface of the hepatocyte (Figure 1) [10].

This low-affinity binding creates the conditions for a high-affinity interaction between a specific domain of HBV envelope and the sodium taurocholate co-transporting polypeptide (NTCP), which serves as a functional receptor for HBV [11]. NTCP is a bile salt transporter located predominantly on the hepatocyte basolateral membrane. Probably, the same co-transporter has a main role also in the interaction with hepatitis D virus (HDV) [12]. These interactions are followed by the endocytosis of the virion, which enters the hepatocytes favoring the cytoplasmic release of the HBV nucleocapsid and its transport to the nucleus [13]. The viral genome is in the form of relaxed circular DNA (rcDNA), but, once released from the nucleocapsid, it is converted into covalently closed circular DNA (cccDNA) by some host DNA repair systems, including tyrosyl-DNA-phosphodiesterase 2 (TDP2) and polymerase kappa (Pol-K) [14,15]. cccDNA is a much more stable version of the viral genome, comparable to a small chromosome. Spliceosome associated factor 1 (SART1) has been recently identified as host factor able to inhibit HBV cccDNA transcription and as potential therapeutic target [16]. cccDNA encodes for six RNAs, which leave the nucleus and produce structural and non-structural viral proteins [17]. In particular, cccDNA acts as a template for the transcription of messenger RNAs (mRNAs) and pregenomic RNAs (pgRNAs). pgRNA and the viral polymerase are rewound together in the viral capsid. Viral replication occurs within these nucleocapsids by reverse transcription of pgRNA. In this process, numerous intermediate products are generated, and their roles are still little known [18]. The result of the reverse transcription is the production of rcDNA (usually present in about 90% of virions) or, less frequently, double-stranded linear DNA (dlsDNA, present in about 10% of virions) depending on whether it occurs or not a specific RNA primer translocation event [19]. At this point, nucleocapsids, containing both rcDNA and dslDNA, can be enveloped and can either be released from hepatocytes as infectious virion or return to the nucleus to amplify the pool of cccDNA molecules [13]. Hepatitis B core-related antigen (HBcrAg) is strongly correlated with the intrahepatic cccDNA reservoir [20]. The stability of cccDNA in nuclei of hepatocytes represents a key determinant of HBV persistence. Overall, HBV has developed a cloaking strategy that avoids recognition by the innate immune system, allowing it to replicate and spread within the liver.

### 2.2. Integration of Viral Genome

HBV DNA integration in the host genome is a central step in the pathogenesis of viral persistence, liver damage, and oncogenesis (Figure 1), although it is not crucial in the viral life cycle and does not produce replicative viruses [21]. It is detectable in hepatocytes, even before the development of liver damage [22,23]. Indeed, its integration occurs in all stages of the disease, starting from the very first days of infection [22,24].

If the reverse transcription process mainly results in the production of rcDNA, in a minority of cases, it determines the production of virions containing dlsDNA (Figure 1) [19]. The rate of production of rcDNA or dlsDNA can vary in relation to their respective viral infection stages, with the proportion of dlsDNA tending to progressively increase during the development of HBV-related liver diseases [25]. Instead of being converted into cccDNA, the HBV genome present in dslDNA-containing virions can integrate into the host cell genome [19]. Through animal models, Bill et al. [26] demonstrated that viral DNA integration occurs at the level of double-stranded DNA breaks in the host cell genome. Unlike retro-viruses, the integration of the HBV genome occurs without the involvement of viral protein-mediated pre-integration complexes [27]. Instead, the role played by the error-prone non-homologous end joining (NHEJ) DNA repair pathways [19,28] and by the regulatory Hepatitis B virus X (HBx) protein [29] in the integration processes appears crucial. The latter promotes transcription of the extrachromosomal viral genome through the inhibition of the structural maintenance of chromosomes (Smc) complex Smc5/6.

The role of integration of the viral genome into the host genome has not yet been fully elucidated. If cccDNA serves as virally replicative template, some rearrangements make the integrated form of HBV unable to perform replicative functions [30]. Indeed, although cell lines derived from integrated HBV DNA are able to express functional HBsAg, they are unable to support viral replication and produce infectious viruses [19]. It has been hypothesized that the integration of HBV DNA into the host cell genome may play a role in the pathogenesis of liver damage and especially in the mechanisms of viral persistence and carcinogenesis. In particular, HBV integration is a significant source of HBsAg expression during chronic infection [31]. Indeed, HBsAg seems to be expressed not only by cccDNA, but also by HBV DNA integrated into the host genome, which was the dominant source in hepatitis B e antigen (HBeAg)-negative infections. Since high levels of circulating HBsAg correlate with virus-specific tolerance [32] and HBV integration [20], this source of HBsAg could represent a viral strategy to maintain chronicity in the presence of host immune-surveillance [31]. In fact, a high level of HBsAg secretion could have an immunosuppressive effect on one hand and, on the other, act as a decoy for the antibody response, altogether allowing the virus to escape immunological control [33].

The integration of the viral genome into that of the host cell would also have a significant role in the mechanisms of oncogenesis, discussed in the next section.

Since integration processes occur in an early phase of viral infection [22], inhibition of reverse transcription by nucleoside analogues does not appear to impact viral integration into the host cell genome [24,34]. Tu et al. [24] tried to identify some points where it is possible to intervene to prevent integration, using different classes of drugs. Surprisingly, Myrcludex-B (Myr-B, an NTCP-inhibitor) was the only drug that demonstrated a significant reduction in DNA integration, hindering virion entry into the hepatocyte.

### 2.3. HBV and Host Immune System

The interactions between HBV and the host’s immune system play a crucial role both in the possibility of viral clearance after acute infection (or vice versa in infection chronicization) and in development of liver damage. The outcome of most infections is strongly determined by the effectiveness of the HBV-specific adaptive immune cell response.

The immune response to virus contact is different in relation to the age of the host and the competence of the immune system. Over 95% of immunocompetent subjects who contract HBV in adulthood develop a self-limited infection. After the acute phase, the immune system effectively eliminates the virus. Conversely, most infections acquired in infancy or early childhood become chronic [35]. In these processes, the adaptive immune response (CD4+ and CD8+ T cell responses, as well as neutralizing antibodies) is significantly more involved than the innate one [36]. In particular, HBV-specific CD8+ T cells are the main effectors of viral clearance in cases of infection resulting in healing through killing of infected hepatocytes and production of antiviral cytokines (interferon-γ, IFN-γ and tumor necrosis factor, TNF) [37]. HBV-specific CD4+ T cells instead act by inducing and favoring the persistence of CD8+ T cell and antibody responses [36]. In chimpanzee models, an early HBV-specific CD4+ T cell response was predictive of viral clearance, and the depletion of CD4+ T cells results in HBV persistence [38]. Viral persistence and the development of chronic liver injury would reflect the dysregulation of these adaptive immune responses [33,39]. Furthermore, the expression of HBsAg by the viral genome integrated into that of the host is likely able to trigger dysfunctional T cell responses and promote immune-mediated liver injury [36].

In a pioneering study, Wieland et al. [40] showed that, in an early stage of infection, there is no robust host response through induction of CD3, IFN-γ, or 2′5′ oligoadenylate synthetase (2′5′ OAS) mRNA, suggesting that viral infection is not detected by the host immune system at an early stage. The authors were also able to demonstrate the limited role of CD4+ cells (whose depletion did not seem to change the natural history of acute infection) and, conversely, the importance of CD8+ cells (whose depletion prolonged the duration of the infection and delayed the viral clearance) [41]. These data are in contrast with what was subsequently highlighted by Asabe et al. [38], who indicate that the CD4+ T cell response is crucial in the initial stages of the infection. Indeed, early CD4+ T cell depletion would result in viral persistence, whereas no impact on infection course was demonstrated for CD4+ T cell depletions obtained six weeks after inoculation. With respect to the CD4+ T cell response, robust data demonstrate the crucial role in viral clearance of CD8+ T cells as major players in the adaptive immune system. Isogawa et al. [42] used transgenic mice to show that, although there is a rapid expansion of HBV-specific CD8+ T-cells in response to infection, they do not rapidly differentiate into effector cells. Thus, an adequate production of IFN-γ and the formation of an immune memory are lacking. Recognition of HBV antigen by naïve CD8+ T cells initiates transcriptional and chromatin changes that result in an overall dysregulated T cell phenotype [43]. For this reason, the cells produced were defined as “exhausted”. The lack of differentiation towards effector cells could be favored by the activation of programmed cell death protein 1 (PD-1), which binds to its own ligand (PD-L1), causing cellular inhibition. Indeed, agonistic anti-CD40 antibodies are able to inhibit PD-1 induction and restore T cell effector function [42]. A central role in these processes also seems to be played by interleukin-2 (IL-2). The administration of IL-2 is indeed able to neutralize the dysfunction of HBV-specific CD8+ T cells [43]. Although the mechanisms by which this occurs are still unproven, during acute HBV infection, high local IL-2 levels may promote an efficient HBV-specific CD8+ T cell response. Since the depletion of CD4+ T cells seems to prevent effective priming of CD8+ T cells and favor the persistence of the infection [38], it is plausible that CD4+ T cells could be a possible source of IL-2 [36]. However, the response of HBV-specific CD4+ T cells is not detectable before the peak of viraemia, when the virus has already infected most of the hepatocytes [38]. Thus, the late triggering of the CD4+ T cell response would not be able to support the development of quantitatively and qualitatively effective CD8+ T cells, favoring viral persistence. Recent evidence also underlines the role of the JAK/STAT system and bone marrow stromal antigen 2 (BST2), a key gene for the production of IFN induced by cells that express CD40 [44]. Similar pathways are those involving cytotoxic T-lymphocyte associated antigen 4 (CTLA-4), T-cell immunoglobulin, and mucin domain-containing protein (Tim-3) [45,46,47,48], making the modulation of the adaptive immune system one of the major targets for future therapeutic approaches.

The effectiveness of the adaptive response and the potential development of a chronic infection depend on the complex interaction between viral and host-related factors. “Tolerance” mechanisms explain the relationship between HBV and adaptive immune system and the virus’ ability to cause chronic disease and are inherent in the physiological behavior of the intrahepatic immune environment. In fact, in the liver, the antigen presentation (not only related to HBV infection), if modest, can determine T-cell inactivation, as well as tolerance and apoptosis of immune cells (lymphocytes, natural killer, NK and dendritic cells, DCs) [49]. Thus, intrahepatic presentation of the antigen by itself triggers negative regulatory signals that prevent functional differentiation of naïve CD8+ T cells. These mechanisms fulfill the need to maintain immunological silence to harmless antigenic material in food. The silencing of T-cell response could explain the tolerance towards numerous pathogens. In the setting of HBV infection, tolerance mechanisms could be related to the intensity of antigen presentation. In particular, a strong viral antigenic stimulus is necessary for an adequate T-cell response to be established, while a slow and long-lasting presentation can lead to inadequate immunity. Indeed, robust CD8+ T cell responses are required for the clearance of HBV. Viral genetic variation and type I IFN signaling determine the magnitude of HBV-specific CD8+ T cell responses by regulating the initial antigen expression levels [50]. Excessive inhibition of HBV-specific CD8+ T cell responses induced by type I IFN signaling could, therefore, favor viral persistence. However, the correlation between intensity of antigen presentation and tolerance mechanisms has not been demonstrated in other studies [43].

Some viral components are believed to play a role in promoting tolerance of the immune system towards HBV. HBeAg could favor HBV chronicity by functioning as an immunoregulatory protein, playing a central role in the persistence of infection [51]. In particular, in models of horizontal transmission (from mother to child), HBeAg appears to affect hepatic macrophages and attenuate the HBV-specific CD8+ T response [52]. Similarly, HBsAg could also favor immune tolerance mechanisms. Elevated levels of circulating HBsAg [36] and/or a long duration of HBsAg exposure [32] have been shown to negatively influence the responses of HBV-specific B and T cells. HBsAg could induce a tolerogenic phenotype both in DCs, whose action is central in the induction of T-cell responses, as well as in monocytes/macrophages [53,54]. In apparent contrast to this hypothesis, however, the dysfunctional immune response would not tend to return to normal after HBsAg clearance [32,55]. In addition to the role played by viral antigens (HBeAg and HBsAg), it is assumed that the presence of specific HBV mutations can influence the immune response against the infection [36]. One potential source of such mutations involves the family of APOBEC3 (apolipoprotein B mRNA-editing catalytic polypeptide-like 3) deaminases, which has demonstrated notable relevance, as it is able to catalyze mutations in both pathogen and human genomes [56]. During chronic HBV infection, host APOBEC3 enzymes can determine both an increase and reduction of these mutations in relation to the overexpressed antiviral factor [57]. However, some data indicate that, although the CD8+ T cell response may be inhibited by mutations in the viral epitopes, a preferential selection of T cells able to overcome the inhibitory effect of such mutations may occur during chronic infection [58].

Although it does not play a predominant role, the innate immune system also contributes to defense mechanisms against HBV, in particular through interferon-λ (IFN-λ) and NK and natural killer T cells (NKT) [59]. Notably, IFN-λ and IFN-λ-stimulated genes (ISGs) are induced in primary infection [60], resulting in the inhibition of viral replication [61]. Other types of interferon have a role in the suppression of HBV replication, such as IFN-α/β and IFN-γ, which are produced by different types of both parenchymal and non-parenchymal cells, such as NK and NKT [62,63,64]. Some data suggest that tool-like receptors (TLRs) play a central role in the activation of these cells [65]. In animal models, TLR7 agonist was able to activate NK, NKT, and T-cells with a consequent suppression of viral replication [66]. TLR8 can activate NK CD56 bright cells and mucosal-associated invariant T (MAIT) cells, which can produce a large amount of IFN-γ [67]. Finally, an interesting series of studies links TLR9 to improved viral clearance through the formation of intrahepatic myeloid-cell aggregates. In mouse models, these stimulate the local proliferation of CD8+ T cells, enhancing the immune response towards the infection [68]. Subsequently, it was observed that the use of TLR9 agonists, through the same pathway, are able to reduce the growth of the liver tumor [69]. Unlike other TLRs, TLR2 appears to support, rather than counteract, viral persistence [70]. However, HBV has evolved strategies to counter TLRs responses, thus limiting adaptive immunity and facilitating viral persistence [65]. In fact, the poor innate immune response following HBV infection suggests that the virus is able to escape these mechanisms and alter type I IFN immune responses in hepatocytes [71]. In particular, both HBV DNA polymerase [72] and HBx protein [73] could inhibit the induction of IFN-β, whereas HBeAg [74] could suppress the expression of TNF-α in peripheral blood mononuclear cells. Furthermore, Li et al. [70] showed that some viral antigens (especially HBcrAg) could favor HBV persistence by suppressing the response of CD8+ T cells and upregulating the expression of TLR2 in liver Kupffer cells. Finally, the NK cell-mediated response is also impaired during chronic HBV infection. Indeed, NK cells appear unable to ensure adequate production of IFN-γ and, consequently, to mediate cytotoxicity [75].

For the reasons discussed, chronic hepatits B could be considered as a disease in which CD8+ T cells, although unable to eliminate the infection, escape tolerance mechanisms and attack hepatocytes in an attempt to eliminate HBV supporting long-term immuno-pathological responses without ever achieving viral clearance [39]. Sustained recognition of HBsAg derived from cccDNA or integration of the viral genome into the host genome is the mechanism that mediates chronic liver injury induced by CD8+ T cells [76]. Therefore, HBV is able to interact with the immune system in order to make it less efficient, thus favoring chronicity. CD8+ T cell-induced hepatic necro-inflammation and consequent hepatocellular regeneration are responsible for the development of liver cirrhosis and HCC during chronic HBV infection.

## 3. HBV-Host Interactions in Oncogenesis

**Figure 2:**
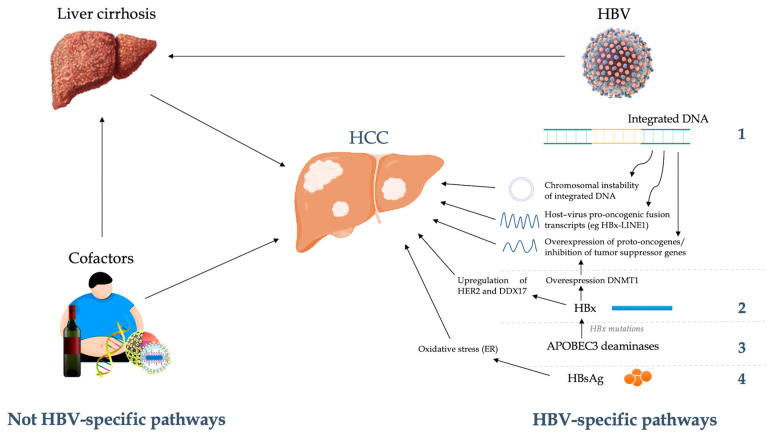
HBV and host interactions in hepatocarcinogenesis.

The mechanisms of persistent necro-inflammation and liver regeneration induced by CD8+ T cells following the continuous antigenic stimulation by HBV are crucial in determining random genetic injury and abnormal repair functions, which lead to liver cirrhosis and HCC (Figure 2) [77]. Compared with non-cirrhotics, HBV-related cirrhotic patients show a 31-fold increased risk of HCC [78]. Injury cofactors (e.g., coinfection with HDV, alcohol consumption), comorbidities (e.g., diabetes mellitus), or gene polymorphisms (e.g., *PNPLA3* or *DEPDC5* polymorphisms) can accelerate the progression of liver fibrosis and the development of neoplasms [79,80,81,82]. These oncogenic mechanisms link HBV infection to other etiologies of chronic liver damage (viral, metabolic, exotoxic, autoimmune) [77,83,84].

Beyond these oncogenic pathways related to the development of chronic liver injury, the complex interactions between HBV and host can per se be a cause of HCC, regardless of the development of liver fibrosis and cirrhosis. In particular, the altered expression of procarcinogenic genes induced by viral integration into the host genome and/or the expression of HBV-derived procarcinogenic proteins have been proposed to directly favor carcinogenesis [85]. Furthermore, chromosomal aberrations and epigenetic changes, and the consequent dysregulation of cell signaling pathways, complete the picture of the mechanisms involved in the development of HBV-related HCC.

By regulating its gene expression, HBV genome integration is a key step in HBV-induced hepatocarcinogenesis (Figure 2) [86,87]. Indeed, the integration of HBV DNA into the host cell genome has shown a close association with the risk of HCC. As proof of this, the frequency of genomic integration of the virus is significantly higher in tumor tissues than in non-tumor ones [88]. Up to 75–90% of HBV-related HCC show HBV DNA integration [89,90]. Although its role in promoting carcinogenesis is established, the mechanisms that correlate integration to oncogenesis are less understood. Some host–virus fusion transcripts derived from the integrated viral genome probably are able to induce oncogenesis through several pathways, including inhibition of apoptosis, direct stimulation of hepatocyte expansion, and the induction of stem-cell like properties [89,91]. Overall, it can be hypothesized that the pro-oncogenic capacity of viral genome integration is due to overexpression of proto-oncogenes and/or inhibition of tumor suppressor genes, the expression of integrated mutant viral protein, and the chromosomal instability of integrated DNA [92,93]. There are some differences related to the integration between non-tumor and tumor cells and these could explain how the interaction between virus and host determines the pathogenic fate of the infection. Although, normally, HBV does not show any major favored sites of integration [94], within tumor cells, such integration is greater at the level of cancer associated genes or central genomic segments in the cell’s life cycle, probably due to selection towards integration events that induces cancer progression [19,20,28,95]. If gene integration occurs in some key regions, the activation of proto-oncogenes or the inhibition of tumor suppressor genes may follow [89,92].

The recurrently targeted genes (RTGs) potentially involved in genomic integration processes are numerous [96,97]. However, Péneau et al. [98] analyzed the characteristics of the HBV integrations involved in hepatocarcinogenesis, showing that, in HCC tumor cells, the genomic integration occurs more frequently at the level of three specific genes: telomerase reverse transcriptase (*TERT*), cyclin E1 (*CCNE1*), and *KMT2B*. The same authors also highlighted how a high degree of viral genomic integration within the HCC correlates with an earlier onset of the tumor and represents a significant negative prognostic factor [98,99]. The *TERT* gene represents, by far, the most involved factor in the mechanisms of viral integration and carcinogenesis [98]. In HBV-related HCC, *TERT* was identified as the most frequent site of HBV integration [100]. Insertional mutations develop more frequently (up to 35% of HCC) at the level of its promoter, resulting in an overexpression of telomerase and consequent conservation of telomere length, with inhibition of cellular senescence and promotion of tumor cell growth [24,99,101]. HBV integration at the TERT promoter is associated with more aggressive tumor behavior [99]. ETS transcription factor 4 (ELF4) is able to activate telomerase and regulate HBV gene transcription. Being able to inhibit *TERT* activation in tumor cells, the knockdown of ELF4 could represent a new therapeutic target in HCC with *TERT* promoter integration.

Integration sites, corresponding to mixed-lineage leukemia 4 (*MLL4*), are also associated with the generation of solid tumors [102,103]. However, the interaction on these genes was found in only 10–15% of HCCs, and they seem to be involved exclusively in a late phase [104]. Therefore, the integration of the viral genome at these sites could favor the persistence of oncogenic mechanisms, rather than being their trigger. In this context, the role played by open reading frames (ORFs) appears crucial. Due to structural rearrangements, ORFs present in the integrated viral genome are frequently altered, with the exception of the HBsAg ORF, which maintains the binding to its promoter intact. On the other hand, some HCCs express transcripts containing the HBeAg/HBcrAg ORFs, and this probably occurs due to active upstream cellular promoters of the integration site [105,106].

As mentioned, in addition to overexpression of proto-oncogenes and/or inhibition of tumor suppressor genes, viral genomic integration can determine the development of HCC through the production of functional virus–host transcript fusions [107]. The production of functional fusion transcripts is strictly dependent on the site and direction of integration and may result in the activation of pathways associated with cell transformation or promoting telomerase overexpression [92]. For example, the fusion gene between the long interspersed nuclear element 1 (*LINE1*) and *HBx* transcribes for HBx-LINE1. The latter is detectable in 23% of HCCs and is associated with worse prognosis, as it would favor tumor progression by activating the Wnt/β-catenin signaling pathway [108].

Beyond the accumulation of genetic injury and abnormal repair functions following the development of liver cirrhosis and the mechanisms of genomic integration, other factors are related to the risk of hepatocarcinogenesis during chronic HBV infection. Several data support a possible direct role of HBsAg in the induction of carcinogenesis (Figure 2). Indeed, high levels of HBsAg were associated with a greater risk of developing HCC, even in patients with suppressed viremia after treatment with nucleos(t)ide analogues [109,110]. Liu et al. [111] showed that the lifetime cumulative incidence of HCC is significantly higher in HBsAg positive patients than in HBsAg negative ones (6.8% vs. 4.0%, respectively) with the same viraemic suppression. Furthermore, patients with HBsAg greater than 1000 IU/mL show a risk of developing HCC 3.8 higher than HBsAg-negative patients. Two mutations in HBsAg have been correlated to the development of HCC. Specifically, two deletions in the pre-S1 and pre-S2 regions were found in the so-called ground glass hepatocytes (GGHs), a pattern recognized to be similar to a precancerous lesion [112,113]. Pre-S1 and Pre-S2 mutants promote progression to HCC due to dysregulation of apoptosis [114,115]. The accumulation of unfolded or misfolded surface proteins causes stress in the endoplasmic reticulum (ER), resulting in reactive oxygen species (ROS) production, oxidative stress development, and DNA damage [116,117].

HBx is also an important actor in the direct oncogenic effect of HBV (Figure 2) [118]. Viral regulatory protein HBx is crucial in the modulation of several cellular and viral signalling processes [119]. A multitude of mechanisms linking HBx to cell cycle and apoptosis dysregulation have been proposed. The ability of HBx to regulate methylation by stimulating the expression of DNA methyltransferase 1 (DNMT1) can lead to the silencing of tumor suppressor genes, as well as favor the creation of zones of instability that are a preferential site for integration [120]. A large number of genes and pathways have been associated with the action of HBx, such as that of p53, ERK–INK4a–RB, HER2, and RAS–RAF–MAPK [121,122,123]. HBx seems to determine HER2 upregulation, favoring growth and migration of HCC cells [23]. Furthermore, HBx seems to be able to induce overexpression of DEAD-box RNA helicase 17 (DDX17) [118]. The latter promotes HBV replication, and its overexpression has been associated with a higher risk of HBx-mediated HCC metastasis. Furthermore, variants of HBx (for example: C-terminally truncated HBx) mutated following viral integration are found in nearly half of HBV-related HCC and could favor hepatocarcinogenesis and metastasis [124]. In this regard, the role of previously cited family of APOBEC3 deaminases appears relevant. If, on one hand, APOBEC3 acts by countering HBV infection through suppression of viral gene transcription and replication, on the other hand, it can induce HBx mutations, giving infected hepatocytes a selective clonal growth advantage [125]. Moreover, aberrant deaminase activity of APOBEC3 may favor genomic instability and lead to HCC development.

Finally, gene mutations or epigenetic changes can alter gene expression and promote tumor occurrence, progression, and metastasis [92,107]. Epigenetic changes secondary to HBV infection are in fact able to directly influence the processes of transcription, translation, and gene expression, affecting the genomic stability of the hepatocyte and favoring the expression of cancer-related genes [126].

## 4. Therapeutic Implications

The treatment of HBV infection is still an ongoing challenge [127]. Beyond the poorly effective interferon (IFN) burdened by numerous side effects, treatments currently available (nucleos(t)ide analogues, NUCs) are able to effectively suppress viral replication, limiting the activity of HBV polymerase with extremely low resistance rates [128]. However, they are rarely able to promote complete recovery, i.e., loss of HBsAg with or without seroconversion to anti-HBs antibody [129]. Furthermore, even when seroconversion occurs (spontaneous or induced by IFN or NUCs), to date, there are no therapeutic strategies capable of obtaining a permanent elimination of all HBV forms, in particular of the cccDNA and integrated HBV DNA [130]. In fact, the complex interactions established early between virus and host make it difficult to obtain a therapy able to definitely eradicate HBV. A definitive cure from HBV infection should therefore provide for the destruction of the cccDNA and the integrated viral genome [131]. For these reasons, alternative therapeutic strategy targeting steps in the HBV life cycle other than its replication are being evaluated.

Many studies are focusing on the interaction with the NTCP receptor in order to prevent the entry of the virus into the cell (Table 1) [132]. In this regard, the most promising molecule seems to be myrcludex-B, a NTCP-inhibitor currently indicated for the treatment of HBV/HDV coinfection. In early phase IIa studies, myrcludex-B, in combination with peginterferon α-2a (PegIFNα-2a), has been shown to significantly reduce the level of HBV DNA, HDV RNA, and ALT [133]. Unfortunately, its action on the reduction of HBsAg appears to be very limited [134].

Anti-HBsAg monoclonal antibodies are under evaluation. In HBV-transgenic mice, single-dose administration of a novel monoclonal antibody E6F6 seems able to suppress the levels of HBsAg and HBV DNA for several weeks [135]. However, since CD8+ T cells play a crucial role in inducing viral clearance and data indicate that HBsAg clearance does not determine the recovery of an effective T cell response [32,52], treatment with anti-HBsAg monoclonal antibodies may only be able to reduce the cccDNA pool, without improving HBV-specific CD8+ T cell responses and favoring definitive recovery from infection [36,130].

Accordingly, modulation of the host’s immune system should represent an important target in viral eradication strategies. Although the first studies date back to the 1990s, currently no therapy is yet validated and many sectors that seemed promising have turned out to be disappointing. Pioneering studies have been performed using IL-2 [136], IL-12 [137], Thymosine-alpha-1 [138,139], Levamisole alone or in association with IFN-α [140]. All of these showed some ability in reducing HBV DNA and improving hepatic cytolytic activity, but failed to show long-term efficacy. According to the role of viral antigens in the infectious process, immunomodulation linked to these components could play a significant role in therapy. Since the ‘90s, attempts have been made to demonstrate the usefulness of therapeutic vaccines based on viral antigens. The use of HBsAg-based vaccines or anti-HBs complexes has proved unsatisfactory [141,142,143]. Similarly, combinations of vaccine with antiviral therapy did not demonstrate superiority over antiviral therapy alone [144,145,146]. Finally, a nasal therapeutic vaccine (NASVAC) containing both HBsAg and HBcAg has demonstrated safety and efficacy, obtaining significantly higher rates of HBeAg seroconversion, HBV DNA negativity and ALT normalization compared to Peg-IFN [147,148,149]. At the 2 [150] and 3 years [151] follow-up after the end of treatment, long-lasting efficacy was confirmed in the reduction of HBV DNA and in ALT normalization. No impact was described on seroconversion rates.

In the field of immunomodulation, another research line concerns the use of monoclonal antibodies inhibiting the PD-1/PD-L1 pathway. As previously discussed, the lack of differentiation of CD8+ T cells into effector cells (a key step in immune tolerance phenomena) could be determined by the activation of PD-1 and through the binding of its ligand PD-L1 [42,45]. In woodchuck model of HBV infection, anti-PD-L1 therapy in association with entecavir (ETV) demonstrated a greater reduction of HBV DNA and HBsAg levels compared to ETV treatment alone, but efficacy was restricted to a minority of animals [152]. Preliminary clinical data on the inhibition of this pathway were obtained from a phase Ib study, that tested the safety and efficacy of nivolumab, a monoclonal antibody inhibitor of PD-1, in combination or not with a HBV therapeutic vaccine, in virally suppressed patients with HBeAg-negative chronic HBV hepatitis [153]. Check-point blockade by nivolumab was well-tolerated and led to HBsAg decline in most patients and sustained HBsAg loss in 1 patient.

Other attempts to potentiate the T cell response have been made. Krebs et al. [154], for example, tested, in mouse models, the safety and efficacy of the enrichment of CD8+ T cells with chimeric antigen receptors (CARs) that bind HBV envelope proteins and activate T cell response. Such engineering of T cells has demonstrated the ability to effectively control viral replication, causing only transient and mild immune-mediated liver injury. Similarly, Kah et al. [155] showed how the administration of T cells engineered to express a HBV-specific T cell receptor (TCR) leads to a progressive reduction of viraemia in absence of persistent organ damage. However, the body of the literature in this field is promising, but it is still very poor.

In the setting of the immune system modulation in order to promote viral clearance, another promising field is represented by the stimulation of innate immunity. Being responsible for initiating intracellular signaling pathways to induce IFNs and other cytokines production, TLRs are recognized as the first line of antiviral immunity. TLRs stimulation can result in suppression of HBV replication. However, HBV has evolved strategies to counter TLR responses, including the suppression of TLR expression and the inhibition of post-receptorial signaling pathways [156]. Since the antiviral treatment for HBV is able to restore the normal antiviral functions of the innate immunity, the activation of TLRs in virally suppressed patients could favor viral clearance. In a phase II, randomized, placebo-controlled study, the administration of Vesatolimod (an oral agonist of TLR-7), in combination with oral antiviral therapy, did not demonstrate significantly higher HBsAg declines than placebo [157].

Stimulators of interferon genes (STING) are modulators of DNA-mediated innate immune activation [158]. Giving their central role in immune activation, they represent potential therapeutic targets during HBV infection. Recently, Li et al. [159] showed, in mouse models, that agonist-induced STING signaling activation in macrophages seems to be able to inhibit HBV replication through epigenetic suppression of cccDNA and mitigate the severity of liver damage through the suppression of macrophage inflammasome activation. These data need to be confirmed in dedicated clinical trials.

The most challenging field is blocking cccDNA formation: blocking this pathway could mean eradicating HBV. Currently, no drugs able to eliminate cccDNA are available or in an advanced stage of study. However, some strategies provide functional silencing by targeting the viral protein HBx (Table 1). In this regard, dicoumarol, an inhibitor of NAD(P)H:quinone oxidoreductase 1 (NQO1), has been shown to have a role in HBV replication, thus affecting HBx protein stability [160]. In fact, endogenous NQO1 acts by protecting HBx protein from proteasome-mediated degradation, and its inactivation significantly reduced the recruitment of HBx to cccDNA and inhibited the transcriptional activity of cccDNA. In mouse models, dicoumarol has shown potent antiviral activity against HBV DNA, HBsAg, HBV RNAs, and HBc protein. Similarly, ribonuclease H (Rnase H) inhibitors have been shown to effectively suppress cccDNA formation (as well as HBV RNA, HBV DNA and HbsAg secretion) in HBV-infected HepG2-NTCP cells [161]. However further studies are needed.

Genetic editing technologies are the most futuristic area in HBV research. Programmable DNA nucleases allow the human genome to be manipulated. Zinc-finger nucleases (ZFNs), transcription activator-like effector nucleases (TALENs), and the clustered regularly interspaced short palindromic repeat/Cas9 (CRISPR/Cas9) system are powerful tools able to bind to and cleave specific DNA sequences (Table 1) [162]. ZFNs were among the first tools to be tested. Cradick et al. [163] used ZFNs to demonstrate the possibility of targeting the episomal viral DNA genome using HBV plasmid transfection models. These engineered nucleases have shown a good ability to reduce viral replication in hepatocytes containing viral genome in the form of integrated DNA or cccDNA [164]. Specifically, the antiviral effect is exerted towards the HBV genome and leads to a reduction in the synthesis of HBeAg, HBsAg, HBcrAg, and pgRNA, as well as a reduction in cccDNA levels. Furthermore, the effect was found to be synergistic to the antiviral power of IFN-α [165].

One of the most promising tools among genetic editing technologies is represented by the CRISPR/Cas9 system. The latter is a platform for efficient gene knockout potentially able to selectively target and cleave conserved regions in the HBV genome, resulting in robust suppression of viral gene expression and replication [166]. In particular, Ramanan et al. [167] have preliminarily demonstrated how this system would be capable of determining a drastic reduction of cccDNA, HbsAg secretion, and viraemia levels. However, due to the ability to induce double-stranded breaks (DSBs), a non-negligible potential in causing harmful mutations in host genome has been suspected [168]. Indeed, DSBs are often repaired by the non-homologous end joining (NHEJ) pathway, frequently resulting in nucleotide insertions or deletions, disruption of gene ORFs, and potentially carcinogenic chromosomal rearrangements [168,169]. In this regard, new CRISPR/Cas9-mediated “base editors” (Bes) have recently been developed [170]. These systems could inactivate the integrated HBV DNA and cccDNA, thus introducing nonsense mutations to specific loci of HBV genome without cleavage of DNA and development of DSBs [171]. Furthermore, the implementation of nanoplatforms has permitted one to lower their immunogenicity and to optimize efficiency, reducing off-target DNA damage [172]. In vivo studies are expected.

**Table 1:** Beyond the nucleos(t)ide analogues: a sample of alternative therapeutic strategies for chronic HBV infection.

Drug Class	Mechanism of Action	Molecule	Ref.	First Author	Year	Study Typology	Sample Size	Results
NTCP-inhibitor	Prevent virus entry into the cell	Myrcludex-B	[133]	Bogomolov	2016	Phase Ib/IIa trial	An amout of 24 patients (HBV/HDV)	Myrcludex-B plus PegIFNα-2a significantly reduce HBV DNA compared to monotherapy. HBsAg levels remained unchanged.
		Myrcludex-B	[134]	Wedemeyer	2019	Phase II trial	An amount of 60 patients (HBV/HDV)	Myrcludex-B 2 mg plus PegIFNα-2a induced HBsAg loss in a substantial proportion of patients
Anti-HBsAg monoclonal Abs	Direct inhibition of HBsAg	E6F6	[135]	Zhang	2016	Pre-clinical trial	HBV-transgenic mice	Single-dose of E6F6 suppressed HBsAg and HBV DNA levels for several weeks
Immuno-modulators	Stimulation of adaptive immune response	IL-2	[136]	Tilg	1993	Phase I + Phase II trial	An amount of 10 patients	No efficacy on HBeAg clearance
		IL-12	[137]	Carreño	2000	Phase I/II trial	An amount of 46 patients	IL-12 reduces significantly HBV DNA levels
		Thymosine-alpha-1	[138]	Iino	2005	72-week multicentre, randomized trial	An amount of 316 patients	Thymosine-alpha-1 therapy is associated with a biochemical and virological response (HBV DNA and HBeAg clearance) in a minority of patients
			[139]	You	2006	RCT	An amount of 62 HBeAg^+^ patients	Thymosine-alpha-1 induce more sustained ALT normalization and HBV DNA and HBeAg loss than IFN-alpha (48% vs. 27%, respectively)
		Levamisole	[140]	Ruiz-Moreno	1993	RCT	An amount of 38 children	No significant differences (biochemical and virological) were observed between Levamisole + IFN and IFN alone groups
	Therapeutic vaccines	NASVAC	[149]	Al Mahtab	2018	Phase III RCT	An amount of 160 patients	NASVAC induced a greater HBV DNA reduction and more frequent clearance rate of HBeAg compared to Peg-IFN
		NASVAC	[151]	Akbar	2021	Phase III RCT	An amount of 160 patients	NASVAC was capable of reducing HBV DNA and normalizing ALT 3 years after the EOT. No reported impact on HBsAg
	Anti-PD-1	Nivolumab	[153]	Gane	2019	Phase Ib trial	An amount of 24 patients	Nivolumab (with or without HBV therapeutic vaccine) was well-tolerated and led to HBsAg decline in most patients and sustained HBsAg loss in 1 patient
	Enrichment of CD8+ T cells with CARs	N/A	[154]	Krebs	2013	Pre-clinical trial	HBV transgenic mice	Engineering of T cells were able to effectively control viral replication
	T cells engineered with HBV-specific TCR	N/A	[155]	Kah	2017	Pre-clinical trial	HBV transgenic mice	T cells engineered to express a HBV-specific T cell receptor leads to a progressive reduction of viraemia in absence of persistent organ damage
	TLR-7 agonism	Vesatolimod	[157]	Janssen	2018	Phase II RCT	An amount of 162 patients	Vesatolimod plus antiviral therapy did not demonstrate significantly higher HBsAg declines than placebo
	STING agonism	N/A	[159]	Li	2022	Pre-clinical trial	HBV mouse model	The activation of STING signaling could inhibit HBV replication and alleviate HBV-induced liver fibrosis
cccDNA silencers	NQO1 inhibitors	Dicoumarol	[160]	Cheng	2021	Pre-clinical trial	Humanized liver mouse model	Potent antiviral activity against HBV DNA, HBsAg, HBV RNAs and HBc protein
	Rnase H inhibitors	3 compounds	[161]	Chauhan	2021	Pre-clinical trial	HBV-infected HepG2-NTCP cells	Rnase H inhibitors effectively suppresses cccDNA formation, as well as HBV RNA, HBV DNA and HbsAg secretion
Genetic editing technologies	TALENs	N/A	[164]	Bloom	2013	Pre-clinical trial	HepG2.2.15 cells	TALENs were able to induce disruption of HBV cccDNA
	CRISPR/Cas9 system	N/A	[171]	Yang	2020	Pre-clinical trial	In vitro HBV infection system	CRISPR/Cas9 system were able to modify episomal cccDNA and suppress viral gene expression

Abs: antibodies; ALT: alanine aminotransferase; CARs: chimeric antigen receptors; cccDNA: covalently closed circular DNA; CRISPR/Cas9: clustered regularly interspaced short palindromic repeat/Cas9; EOT: end of treatment; ETV: entecavir; HBeAg: hepatitis B envelope antigen; HBsAg: hepatitis B surface antigen; HBV: hepatitis B virus; HDV: hepatitis D virus; IFN: interferon; IL-2: interleukin-2; IL-12: interleukin-12; N/A: not available; NCTP: sodium taurocholate co-transporting polypeptide; NQO1: NAD(P)H:quinone oxidoreductase 1; PD-1: programmed cell death protein 1; PegIFNα-2a: peg-interferon α 2a; RCT: randomized clinical trial; Rnase H: ribonuclease H; STING: stimulator of interferon genes; TALENs: transcription activator-like effector nucleases; TCR: T cell receptor; TLR: toll like receptor.

## 5. Open Issues and Perspectives in HBV Infection Therapy

In the last decades, important forward steps have been made in understanding the interactions between HBV and host, which are responsible for liver damage, viral persistence, and oncogenesis. At the same time, antiviral drugs (NUCs) that are safe and effective in suppressing replication have been developed, thus switching off liver inflammation and reducing the risk of progression and HCC. A definitive “cure” for HBV infection able to induce not only HBsAg loss, but, above all, to eradicate cccDNA and the viral genome that has been integrated in that of the host, appears to be still far away. [131]. Numerous targeting virus entry molecules (bulevirtide), capsid assembly modulators (CAMs) and viral protein productions and HBV DNA replications (siRNAs and antisense oligonucleotides) are being researched in pre-clinical and clinical trials. The CRISPR/Cas9-mediated Bes system could be a potentially curative promising strategy for chronic hepatitis B capable of permanently silencing both integrated HBV genome and cccDNA, with low risk of host genome rearrangement and carcinogenesis [171,172]. Neverthless, beyond efficient antiviral activity, available evidence shows that the restoration of immune response to HBV seems to be essential. For this reason, immune modulatory therapies able to stimulate immune response (adaptive or innate) and/or counter HBV-induced immune blockade are being evaluated and implemented [173]. If innate immune stimulation strategies (through TLR agonists) [157,174] or HBV-specific T-cell (through therapeutic vaccines) [141,142,143,144,145,146,147,148,149] response or restoration of robust immune responses (through checkpoint inhibitors such as programmed death receptor-1, PD-1, blocking antibodies) [175] have been demonstrated, alone, to reach a partial and not satisfying efficacy, combination therapies (antiviral plus immunomodulators drugs) could represent more promising strategies able to achieve a functional HBV cure. In a recent phase 1b/2a trial, Evans et al. [176] have preliminarily showed a promising effectiveness of triple combination therapy. The association of a therapeutic vaccine (obtained from a chimpanzee adenoviral vector and a heterologous modified vaccinia Ankara boost, MVA-HBV) with low-dose nivolumab (a PD-1 inhibitor) in virally suppressed patients (through NUCs) resulted in greater HBsAg decline than vaccine alone. Additionally, the loss of HBsAg in one patient out of 18 that were treated overall (5.6%) was observed. These preliminary data need to be explored. At the same time, also, cccDNA transcription level assessment methods should be implemented and standardized to evaluate response to treatment. This assessment could need surrogate markers for cccDNA transcription, such as HBV RNA or HBcrAg, which are already under consideration [177,178].

## 6. Conclusions

The complex interactions between HBV and host account for the ability of the virus to escape immunological control and induce chronicity and risk of hepatocarcinogenesis. Indeed, a host’s immune system response is significantly affected by numerous viral components that act to promote immune tolerance and infection persistence. Moreover, the expression of procarcinogenic genes induced by viral integration into the host genome and the expression of HBV-derived procarcinogenic proteins directly favor carcinogenesis, regardless of liver fibrosis development. At the same time, these interactions (HBV–host) make it challenging to obtain definitive viral eradication. The integration of the viral genome and the stability of the cccDNA are the main obstacles to overcome in order to obtain to a functional cure for chronic HBV infection. A definitive cure of the infection must involve elimination both of cccDNA and integrated viral genome genetic editing technologies and/or combination therapies capable of acting at several levels of the virus life cycle (e.g., replication inhibition, antigen reduction, and immune stimulation). These developments represent the most fascinating therapeutic perspectives to date. Thanks to the increasingly profound knowledge of the mechanisms of viral evasion or resistance and the development of advanced technologies, we could be able to win the challenge of eradicating HBV.

## Data Availability

No new data were created or analyzed in this study. Data sharing is not applicable to this article.
